# Nanotechnology for Radionuclide and Heavy Metal Removal in Mining-Impacted Water Systems: Advances, Challenges, and Future Directions

**DOI:** 10.3390/toxics14090819

**Published:** 2026-09-15

**Authors:** Lethabo G. Selala, Thandiwe Sithole, Phoka C. Rathebe

**Affiliations:** 1 Department of Environmental Health, Faculty of Health Sciences, University of Johannesburg, Doornfontein Campus, P.O. Box 524, Johannesburg 2006, South Africa; 222198027@student.uj.ac.za; 2 South African Weather Service, Climate Services, Private Bag X097, Pretoria 0001, South Africa; 3 Department of Chemical Engineering, Faculty of Engineering and the Build Environment, University of Johannesburg, Doornfontein Campus, P.O. Box 17011, Johannesburg 2206, South Africa; nastassias@uj.ac.za

**Keywords:** mining impacted water, radionuclides, heavy metals, nanomaterials, sustainable remediation

## Abstract

Mining activities release radionuclides and toxic heavy metals into aquatic systems, creating long-term risks to water quality, ecosystems, and human health. Conventional remediation technologies often show limited effectiveness in mining-impacted waters due to complex geochemical conditions, including high salinity, variable pH, and co-occurring contaminants. This review evaluates non-graphene nanomaterials as emerging tools for mitigating radionuclide and heavy metal contamination, focusing on magnetic nanoparticles, metal oxides, nanoclays and zeolites, metal organic frameworks (MOFs), and biogenic nanoparticles. Graphene-based nanomaterials are excluded from the present review because they have been comprehensively addressed in a dedicated review previously published by the authors. A comparative evaluation of these nanomaterial classes demonstrates that no single material is universally optimal; rather, remediation performance depends on contaminant speciation, water chemistry, and operational requirements. Emphasis is placed on the mechanistic processes governing contaminant removal, including ion exchange, surface complexation, chemisorption, physisorption, and photocatalytic redox reactions. The role of environmental factors such as pH, ionic strength, competing ions, and natural organic matter in controlling nanomaterial performance is critically assessed. While laboratory studies demonstrate high removal efficiencies, practical implementation remains constrained by nanoparticle aggregation, long-term stability, recovery, and scalability under realistic mining-water conditions. The review highlights composite materials, immobilized nanostructures, green synthesis routes, and hybrid treatment systems as viable pathways for field deployment. This work underscores the importance of mechanistic insight and environmental compatibility in translating nanomaterial-based remediation strategies from laboratory research to sustainable water management solutions in mining-impacted environments.

## 1. Introduction

Mining activities are major contributors to the release of radionuclides and toxic heavy metals into aquatic environments, particularly in areas affected by gold and uranium mining. Gold and uranium extraction generates vast volumes of waste rock and tailings enriched with uranium, thorium, radium, and associated decay products, as well as toxic metals such as lead, arsenic, cadmium, and mercury, which can contaminate groundwater and surface-water systems. Once mobilized, these substances pose long-term threats to drinking water quality, ecosystem stability, and human health, especially in regions where communities rely directly on groundwater sources [1]. Acid mine drainage (AMD) further enhances contaminant mobility by lowering pH and increasing metal solubility [2]. Unlike many organic pollutants, radionuclides remain hazardous through radioactive decay, while heavy metals persist, accumulate in sediments and aquatic organisms, and may biomagnify through the food chain. Concentrations exceeding drinking-water guidelines have been reported in mining-impacted regions, with associated risks including carcinogenic, neurological, and gastrointestinal effects [3,4,5]. Their persistence, mobility, and complex physicochemical behaviour, combined with limited treatment infrastructure in some affected communities, present significant challenges for sustainable remediation [6,7].

Conventional methods for treating radionuclide- and heavy metal-contaminated water include aeration, granular activated carbon (GAC) filtration, chemical precipitation, ion exchange, and membrane-based processes. While these technologies have demonstrated varying degrees of effectiveness, they face significant limitations in mining-impacted settings. Aeration and GAC, for example, can reduce radon levels but require frequent maintenance and generate secondary waste that must be carefully managed [8]. Chemical precipitation and ion exchange are effective for heavy metals but are highly dependent on water chemistry and generate large volumes of sludge, complicating disposal [9]. Membrane technologies such as reverse osmosis offer high removal efficiency but are costly, energy-intensive, and susceptible to fouling, making them unsuitable for widespread use in low-resource communities [10]. These constraints highlight the pressing need for alternative approaches that are not only effective and scalable but also economically and environmentally sustainable.

Nanotechnology has emerged as a promising alternative for addressing the limitations of conventional water treatment methods. Owing to their high surface area, tunable surface chemistry, and unique physicochemical properties, engineered nanomaterials such as carbon nanomaterials, zeolites, and metal oxides exhibit exceptional adsorption capacities and can facilitate catalytic and redox processes for the efficient removal of radionuclides and heavy metals [11,12]. Unlike traditional technologies, many nanomaterials can be functionalized to enhance selectivity, stability, and regeneration potential, thereby reducing operational costs and minimizing secondary waste generation [13]. Recent advancements in green synthesis and hybrid nanocomposite design have further improved the environmental compatibility and scalability of these materials, making them increasingly suitable for deployment in mining-impacted water systems [14,15]. Collectively, these advantages position nanotechnology as a versatile and sustainable solution to the persistent challenges posed by radionuclides and heavy metals in contaminated aquatic environments.

Several reviews have examined the application of nanomaterials in contaminated-water remediation; however, their scopes differ substantially from that of the present review. Baby et al. [11] reviewed carbon nanomaterials for heavy-metal-contaminated water, while Ethaib et al. [12] examined the role of nanomaterials in heavy metal removal from water and wastewater. Karnwal and Malik [13] further evaluated engineered nanomaterials for cleaner water, with emphasis on heavy metal removal, whereas Mathur et al. [15] considered nanomaterials for water remediation primarily in relation to metal(loid) hazards. Collectively, these reviews have largely focused on nanomaterial-based contaminant removal from water generally, rather than on the distinctive geochemical, operational, and socio-economic constraints associated with mining-impacted water systems. Although they provide important foundations, they do not comprehensively integrate multiple nanomaterial classes with contaminant co-occurrence, mining-water chemistry, environmental stability, cost considerations, or field-scale applicability.

The present review addresses this gap by providing a comparative and mechanistic assessment of magnetic nanoparticles, metal oxides, metal–organic frameworks, nanoclays and zeolites, and biogenic nanoparticles for the removal of radionuclides and toxic heavy metals from mining-impacted waters. Graphene-based materials are excluded from detailed consideration because they have been addressed in a dedicated review previously published by the authors. Particular attention is given to how contaminant speciation and mining-water characteristics, including pH, ionic strength, competing ions, and natural organic matter, influence nanomaterial performance. The review further evaluates environmental stability, regeneration, cost, scalability, and laboratory-to-field translation to support the practical application of nanomaterial-based remediation in mining-impacted water systems.

## 2. Literature Search Methodology

### 2.1. Literature Search Strategy

This review adopted a semi-systematic critical review approach to evaluate the application of nanotechnology-based materials for the remediation of radionuclides and heavy metals in mining-impacted water systems. A comprehensive literature search was conducted using major scientific databases, including Scopus, EBSCOhost, ScienceDirect, SpringerLink, Wiley Online Library, MDPI Open Access Journals, and Google Scholar, accessed through the University of Johannesburg Library website. The search focused primarily on peer-reviewed articles published between 2018 and 2026 to ensure the inclusion of recent advances in nanomaterial synthesis, adsorption mechanisms, photocatalytic remediation, and mining wastewater treatment technologies.

**Figure 1:**
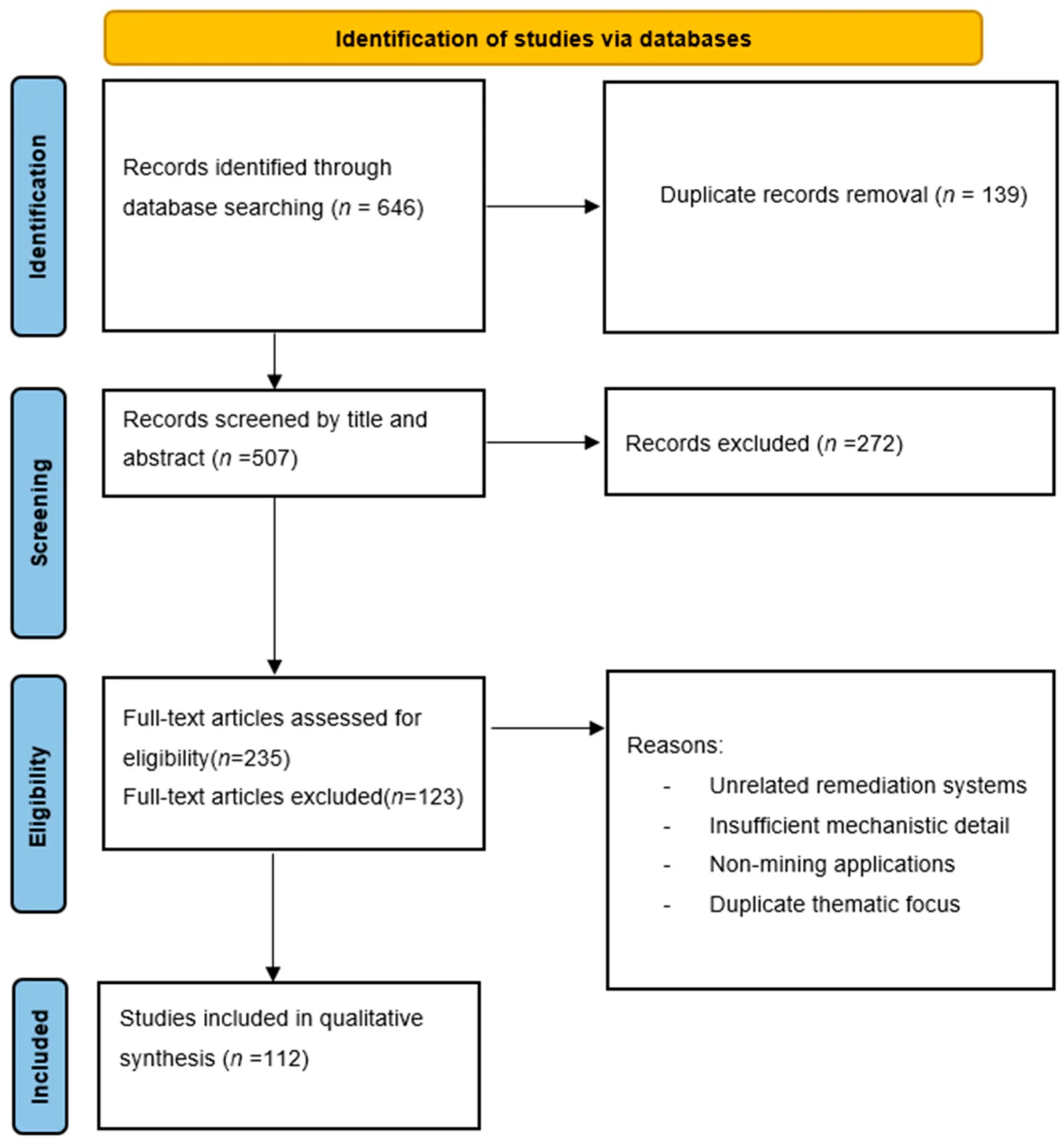
PRISMA-style flow diagram illustrating the literature search, screening, eligibility assessment, and study selection process adopted for this semi-systematic critical review.

The search process employed combinations of keywords and Boolean operators related to radionuclides, heavy metals, mining contamination, and nanotechnology-based remediation. The primary search terms included “radionuclides”, “heavy metals”, “mining wastewater”, “acid mine drainage”, “nanomaterials”, “magnetic nanoparticles”, “metal oxide nanoparticles”, “metal–organic frameworks”, “nanoclays”, “zeolites”, “adsorption”, “photocatalysis”, and “water remediation”. Additional searches were performed using combinations of contaminant-specific terms such as “uranium”, “radium”, “arsenic”, “lead”, and “cadmium” to capture studies relevant to mining-impacted aquatic environments. The overall literature search and study selection workflow adopted in this semi-systematic critical review is presented in Figure 1.

### 2.2. Inclusion and Exclusion

The review considered peer-reviewed journal articles, review papers, and selected technical reports that focused on nanotechnology applications for the removal of radionuclides and heavy metals from contaminated water systems, particularly in mining-related environments. Studies investigating adsorption mechanisms, photocatalytic degradation, ion exchange processes, nanomaterial synthesis, environmental stability, and field-scale remediation potential were prioritized. Emphasis was placed on studies addressing acid mine drainage (AMD), mixed contaminant systems, and realistic environmental conditions relevant to mining-impacted waters.

Studies were excluded if they focused exclusively on non-aqueous remediation systems, unrelated industrial wastewater applications, or nanomaterials without direct relevance to radionuclide or heavy metal removal in mining-associated environments. However, selected studies addressing airborne radionuclide exposure pathways, particularly radon inhalation and associated human health risks in mining regions, were included where relevant to environmental contamination and exposure assessment. Purely graphene-focused studies were excluded from detailed discussion because graphene-based materials are addressed separately in a dedicated review prepared by the authors. Non-English publications, conference abstracts without full-text availability, and studies lacking sufficient experimental, environmental, or mechanistic detail were also excluded.

### 2.3. Data Analysis and Thematic Organization

The selected literature was critically analyzed and organized according to nanomaterial class, dominant remediation mechanism, target contaminants, and practical applicability in mining-impacted water systems. Thematic synthesis was used to compare the performance of magnetic nanoparticles, metal oxides, metal–organic frameworks (MOFs), nanoclays, zeolites, and biogenic nanoparticles under varying environmental conditions, including pH, ionic strength, competing ions, and contaminant mixtures.

In addition to evaluating laboratory-scale removal efficiencies, the review examined key limitations affecting field implementation, including aggregation, regeneration, long-term stability, scalability, and environmental risks associated with nanomaterial deployment. Comparative analysis was further used to identify emerging trends, research gaps, and future opportunities related to hybrid treatment systems, green synthesis approaches, circular economy integration, and sustainable remediation strategies for mining-affected water environments.

## 3. Occurrence of Radionuclide and Heavy Metals in Mining-Affected Waters

### 3.1. Geological and Mining Sources

**Figure 2:**
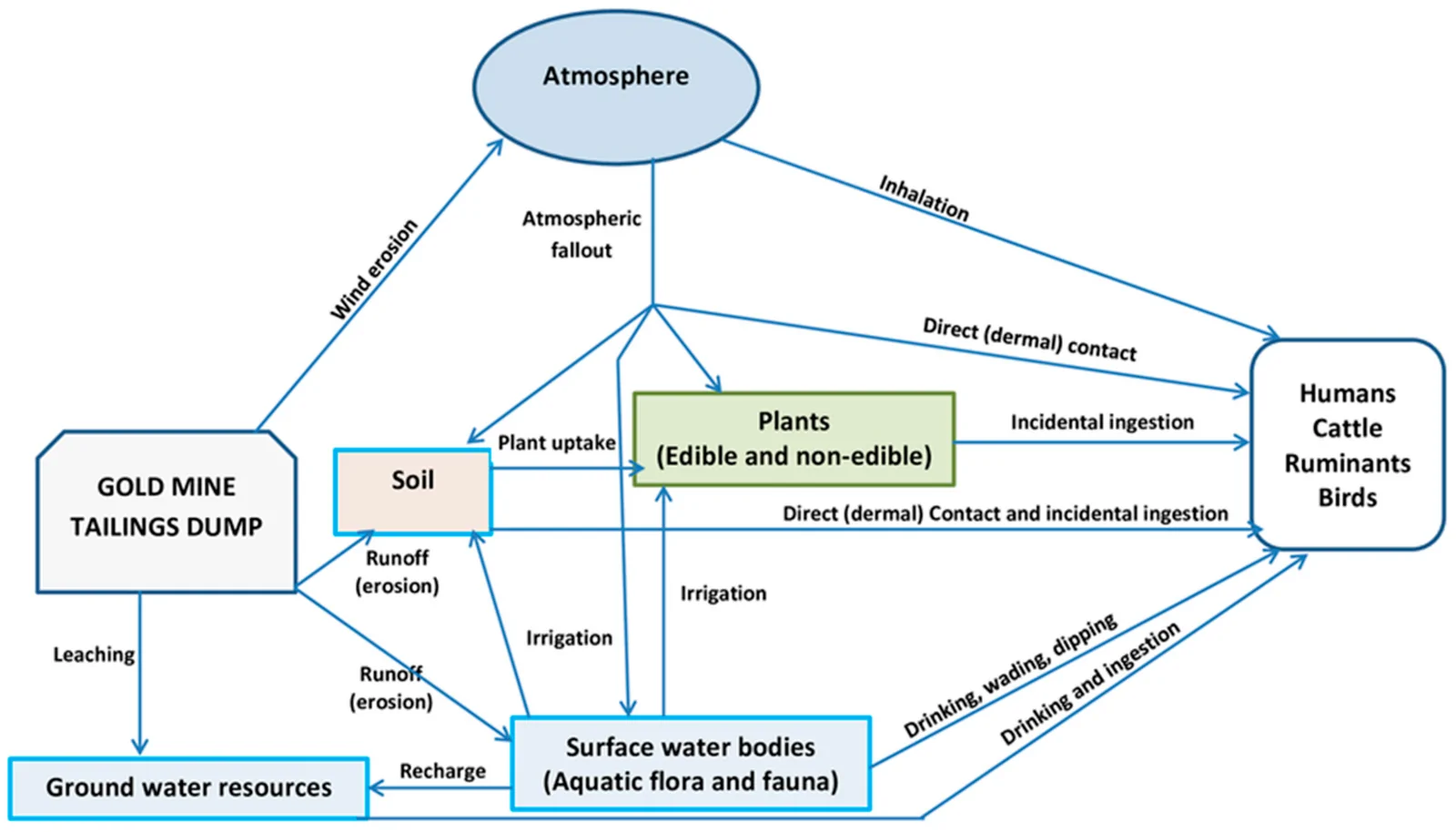
Conceptual illustration of radionuclide and heavy metal release from mining activities, environmental transport pathways, and exposure routes to humans and ecosystems [19].

Radionuclides and heavy metals occur naturally in mineral-bearing geological formations and may enter groundwater through weathering and leaching processes [1,16,17]. Mining substantially increases their release by exposing and concentrating mineralized materials in waste rock and tailings, which become secondary contamination sources through leaching, seepage, and erosion [1,18]. Importantly, the resulting contaminant profile varies with mine type and ore mineralogy. Gold and uranium mining can generate waters containing uranium-series radionuclides together with metals such as arsenic and lead, whereas lead–zinc and polymetallic mining environments are more strongly associated with Pb, Zn, Cd, and related trace metals. Artisanal and small-scale gold mining presents a distinct profile where mercury used during amalgamation may become an important aquatic contaminant [3]. Thus, mining-impacted waters should not be treated as having a uniform contaminant composition; the dominant contaminant assemblage reflects the mineral deposit, extraction practices, and characteristics of the resulting mine wastes. The principal sources, transport pathways, and exposure routes are illustrated in Figure 2.

### 3.2. Environmental Pathways and Regional Contamination Patterns

Despite differences in contaminant profiles, mining regions share several pathways through which contaminants are transferred into aquatic systems. Acid mine drainage (AMD), generated through the oxidation of sulfide minerals, creates acidic conditions that enhance metal mobilisation, while leaching, seepage, rainfall-driven runoff, erosion, and sediment transport facilitate contaminant transfer from mine wastes into groundwater and surface-water systems [20,21,22]. However, the relative importance of these pathways varies according to local geology, mine type, climate, and water chemistry. For example, gold- and uranium-mining environments may exhibit mixed radionuclide and metal contamination [1], whereas lead-mining areas can be dominated by elevated concentrations of Pb and associated heavy metals in wells and surface waters [3,5]. Across these different settings, a common pattern is the mobilisation of multiple contaminants from mineralized wastes into surrounding aquatic systems [5,6]. These similarities and differences indicate that remediation requirements are site-specific and should account for the dominant contaminant profile, mine type, and hydrogeochemical conditions.

### 3.3. Case Studies: South Africa and Other Mining Regions

Radionuclide and heavy metal contamination in mining regions, particularly in South Africa’s Witwatersrand Basin, poses serious environmental and public health risks. Gold and uranium mining in this area has produced extensive tailing dams that continuously leach toxic elements such as uranium, radium, arsenic, and lead into surrounding water systems [23,24]. Groundwater studies have reported uranium concentrations exceeding the World Health Organization (WHO) guideline of 30 µg L^−1^, with some sites recording levels as high as 781.9 µg L^−1^ [25]. More recent evidence from historical mine wastelands in Brakpan, Gauteng, has similarly demonstrated the co-occurrence of heavy metals and naturally occurring radioactive materials in mining-impacted environments [26]. Comparable challenges have been documented globally. In India, elevated uranium levels have been reported in groundwater near mining sites, while artisanal gold mining in Nigeria has led to severe lead contamination of water sources, triggering a severe public health crisis [23,27]. In China and Brazil, acid mine drainage from coal and gold mining has facilitated the mobilization of arsenic, cadmium, and uranium into rivers and reservoirs, extending contamination far downstream [23]. These examples underscore that the combined challenge of radionuclides and heavy metals is not confined to a single region but is a global issue, consistently placing mining-impacted communities at heightened risk of exposure and adverse health outcomes. As summarized in Table 1, concentrations of radionuclides and toxic heavy metals in mining-impacted waters commonly exceed World Health Organization guideline limits by one to several orders of magnitude, underscoring the urgent need for effective remediation strategies.

## 4. Health and Environmental Risks

### 4.1. Radionuclides Exposure Pathways and Risks

Exposure to radionuclides in water systems occurs primarily through ingestion and inhalation, with both pathways carrying substantial health risks. In South Africa, the legacy of gold mining has resulted in elevated levels of uranium, radium, and thorium in groundwater, particularly in the West Rand, where annual effective doses often exceed World Health Organization (WHO) and European Union thresholds, raising cancer morbidity and mortality risks, especially among females due to their longer life expectancy [1,34]. In the Rietspruit catchment, uranium contamination presents a greater chemical toxicity risk than radiological risk, with chemical risk indices reported as high as 37.2, underscoring the need for urgent intervention [25]. Globally, similar concerns have been reported. In Nigeria, radon concentrations in groundwater are generally within safe limits, but exceedances during rainy seasons pose risks, particularly to children and infants [35,36]. In parts of India, uranium levels in groundwater surpass WHO guidelines, with health assessments indicating that chemical toxicity, rather than radiation dose, is the primary concern [37]. Collectively, these findings demonstrate that radionuclide exposure presents both chemical and radiological hazards, with risks varying by contaminant and region. They also highlight the urgent need for continuous monitoring and robust regulatory control to protect communities in mining-impacted areas.

### 4.2. Heavy Metal Toxicity and Bioaccumulation

Heavy metals released from mining activities in South Africa, particularly arsenic, lead, cadmium, and mercury contamination, pose significant health risks due to their persistence and toxicity. Arsenic, a known carcinogen, is frequently mobilized from mining tailings and has been linked to cancers of the skin, bladder, and lungs, as well as cardiovascular disease [38]. Lead contamination, especially in communities near gold and uranium tailings, often exceeds the World Health Organization (WHO) guideline of 0.01 mg L^−1^, raising serious concerns for child health and neurological development [39]. Cadmium, commonly associated with acid mine drainage, contributes to kidney damage and skeletal issues. At the same time, mercury from artisanal mining bioaccumulates in aquatic ecosystems, posing a threat to populations reliant on contaminated water and fish [40]. In the Wonderfonteinspruit catchment, studies have documented heavy metal concentrations in sediments and waters exceeding safety thresholds, indicating long-term exposure risks for surrounding communities [39].

### 4.3. Combined and Cumulative Effects

**Figure 3:**
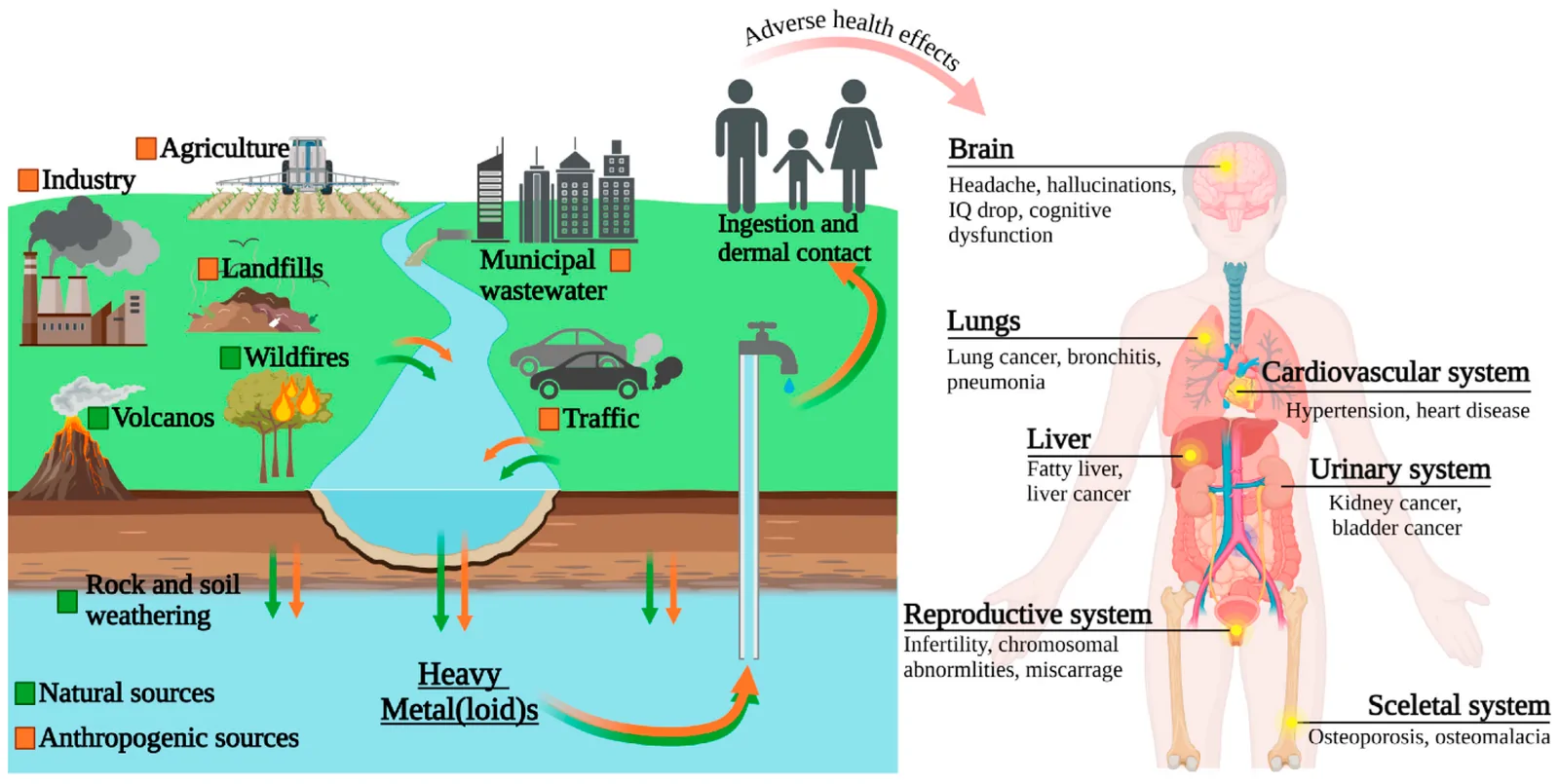
Combined health and ecological risks associated with co-exposure to radionuclides and heavy metals in mining-impacted environment. Orange arrows indicate anthropogenic sources, while green arrows indicate natural sources [41].

Communities in mining-impacted regions of South Africa are often exposed simultaneously to radionuclides and heavy metals, resulting in combined health risks associated with multiple contaminants. Studies in the Witwatersrand Basin show that uranium in water not only poses radiological risks but also acts as a chemical toxin affecting kidney function, while co-occurring metals such as lead and cadmium contribute additional toxicological risks [1,25]. Acid mine drainage further enhances this burden by increasing the mobility of both radionuclides and metals, leading to cumulative exposure through drinking water, soil, and food chains [24]. Research in the Wonderfonteinspruit catchment indicates that hazard indices for multiple contaminants often exceed safe limits, confirming that residents face overlapping risks of cancer, organ damage, and developmental disorders [39]. The proximity of settlements to mine tailings in Gauteng intensifies these exposures, making cumulative effects a pressing public health concern that requires integrated monitoring and remediation strategies. The interconnected human health and ecological risks arising from co-exposure to radionuclides and heavy metals in mining-impacted environments are schematically summarized in Figure 3.

### 4.4. Ecological Risks

Beyond human health, radionuclides and heavy metals from mining activities also disrupt aquatic and terrestrial ecosystems in South Africa. In the Witwatersrand Basin, elevated concentrations of uranium, arsenic, and cadmium have been linked to reduced biodiversity, impaired reproduction in aquatic organisms, and bioaccumulation in fish, transferring risks through the food chain [42,43]. In the Wonderfonteinspruit catchment, long-term accumulation of contaminants in sediments has created chronic exposure pathways for benthic organisms and wildlife that depend on these habitats [6]. Similar impacts are reported in the Crocodile River system, where toxic elements such as nickel, zinc, arsenic, lead, and uranium exceed quality guidelines, threatening aquatic life and water quality [23]. The persistence of contaminants in soil further undermines agricultural productivity near mining areas, posing risks to food security and ecosystem services [44]. Collectively, these findings show that mining-related contamination extends far beyond direct human exposure, weakening ecosystem resilience and endangering the livelihoods that depend on it.

## 5. Limitations of Conventional Treatment Technologies in Mining Context

### 5.1. Aeration, Granular Activated Carbon (GAC), Membranes, and Protection

Conventional technologies commonly applied to treat radionuclide- and heavy metal-contaminated water include aeration, granular activated carbon (GAC) filtration, chemical precipitation, ion exchange, and membrane-based processes. Aeration and GAC are particularly effective for radon removal because of radon’s low solubility and high vapor pressure, which facilitates its transfer from water to air; however, these methods require frequent regeneration and safe disposal of spent media, creating logistical and environmental management challenges [45]. Chemical precipitation and ion exchange can effectively remove metals such as lead, cadmium, and uranium under controlled conditions, but their performance is strongly influenced by pH, ionic strength, and the presence of competing ions commonly encountered in mining-impacted waters [46]. In acid mine drainage (AMD)-affected systems, elevated sulfate concentrations, acidic conditions, and mixed contaminant loads can significantly reduce treatment selectivity and increase sludge production, resulting in high operational and disposal costs. Membrane processes such as reverse osmosis and nanofiltration achieve high removal efficiencies for dissolved radionuclides, metals, and salts; however, their application in mining environments is constrained by membrane fouling, scaling, high energy demand, and expensive maintenance requirements [47]. Although conventional treatment technologies remain valuable for short-term remediation and centralized treatment applications, their technical limitations, secondary waste generation, and poor adaptability to complex mining conditions reduce their sustainability for large-scale deployment in regions such as the Witwatersrand Basin.

### 5.2. Infrastructure and Cost Limitation in Low-Resource Regions

The application of conventional water treatment technologies in mining-impacted regions of South Africa is significantly constrained by infrastructure, cost, and maintenance requirements. Many affected communities are located in peri-urban and rural areas where access to reliable electricity, skilled operators, and waste management systems is limited [24]. High operational costs make advanced methods such as membrane filtration or ion exchange economically unfeasible, particularly when treatment plants must operate continuously to manage ongoing contamination from acid mine drainage. The sludge generated from chemical precipitation and ion exchange also requires safe disposal, yet many municipalities lack adequate infrastructure to handle toxic waste streams, raising the risk of secondary pollution [46]. Furthermore, frequent maintenance needs, such as replacing GAC filters or cleaning fouled membranes, impose additional financial burdens that most low-resource communities cannot sustain [48]. These infrastructure and cost barriers explain why conventional technologies, while technically effective in controlled conditions, often fail to provide sustainable solutions in South Africa’s mining-impacted water systems. The limitations of conventional water treatment technologies in mining-impacted systems are summarized in Table 2.

### 5.3. Rationale for Alternative Treatment Technologies

The shortcomings of conventional water treatment methods in mining contexts underscore the urgent need for technologies that are both effective and adaptable to local resource constraints. In South Africa’s mining regions, particularly the Witwatersrand Basin, treatment systems must address not only the chemical complexity of water, often containing mixtures of radionuclides, heavy metals, and acid mine drainage (AMD) but also the socio-economic realities of affected communities. Sustainable alternatives should minimize secondary waste generation, reduce operational costs, and be deployable across multiple scales, from household point-of-use systems to municipal treatment facilities. Consequently, there is growing interest in remediation technologies capable of maintaining high selectivity, stability, and operational efficiency under the harsh physicochemical condition’s characteristic of mining-impacted waters. Recent advances in materials science, particularly nanotechnology-based approaches, offer promising alternatives by combining high removal efficiencies, enhanced selectivity, regeneration potential, and reduced environmental impact. Developing such targeted, scalable, and sustainable technologies is critical for mitigating the persistent risks posed by mining-related contamination and safeguarding water security in vulnerable communities.

## 6. Nanotechnology-Based Remediation Strategies

**Figure 4:**
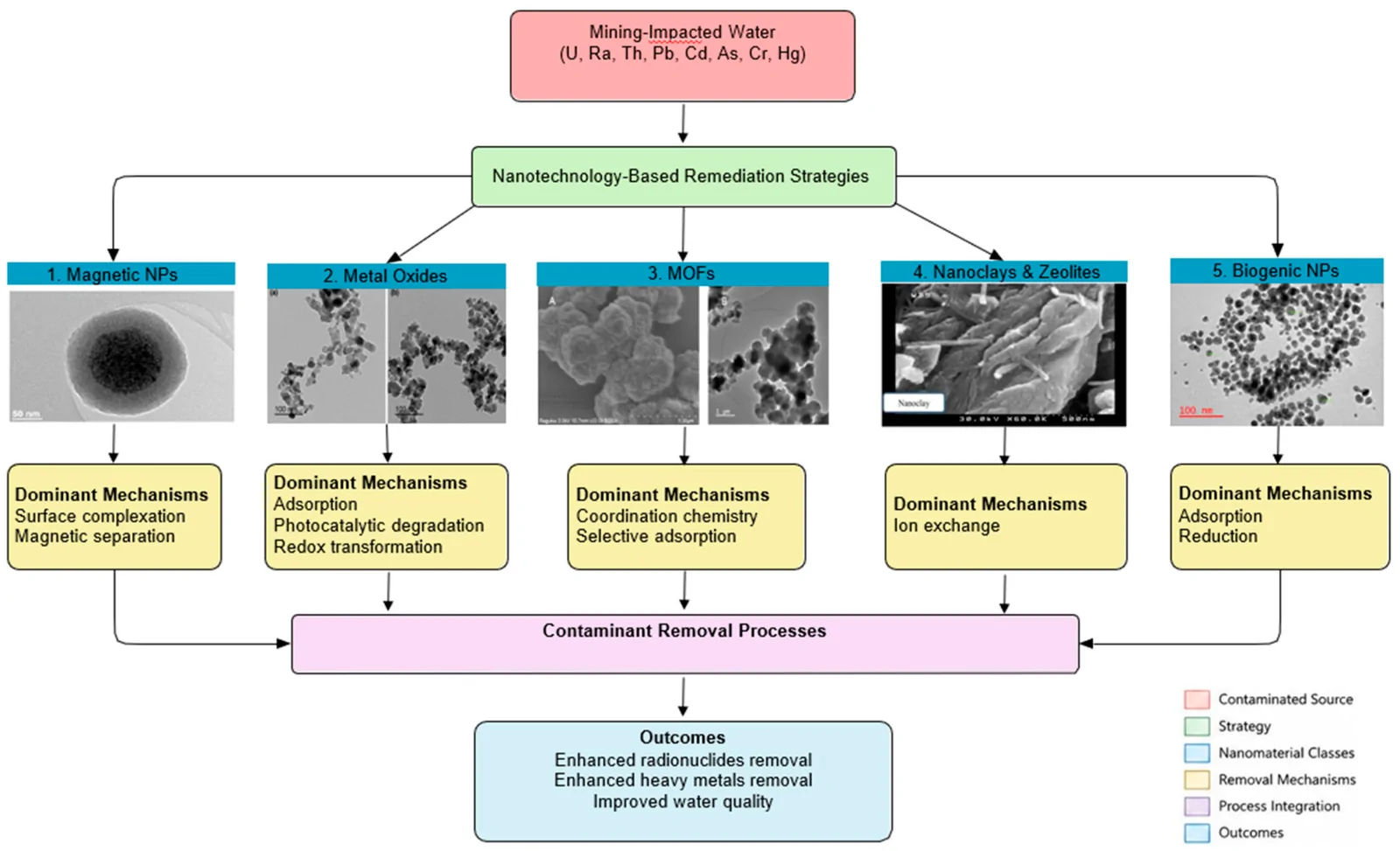
Conceptual framework illustrating the principal nanomaterial classes, dominant removal mechanisms, and treatment outcomes associated with nanotechnology-based remediation of radionuclide- and heavy metal-contaminated mining water.

Nanotechnology has attracted growing interest as an alternative approach for treating radionuclide- and heavy metal-contaminated water in mining regions. This section evaluates the material-specific properties and remediation performance of magnetic nanoparticles, metal oxides, metal–organic frameworks (MOFs), nanoclays and zeolites, and biogenic or green-synthesized nanoparticles. Attention is given to their target contaminants, reported treatment performance, operational conditions, regeneration potential, limitations, and practical applicability in mining-impacted water systems. An overview of the principal nanomaterial classes, their dominant remediation mechanisms, and the corresponding treatment outcomes is presented in Figure 4.

### 6.1. Magnetic Nanoparticles (Fe_3_O_4_, CoFe_2_O_4_)

Magnetic nanoparticles (MNPs), particularly magnetite (Fe_3_O_4_) and cobalt ferrite (CoFe_2_O_4_), are among the promising nanomaterials for water treatment due to their high surface area, strong adsorption capacity, and the advantage of easy recovery using magnetic fields. Fe_3_O_4_ nanoparticles exhibit a strong affinity for radionuclides and heavy metals such as uranium, arsenic, and lead through surface complexation and electrostatic interactions, with optimal removal typically achieved under near-neutral pH (5–7) conditions [53]. In contrast, CoFe_2_O_4_ nanoparticles demonstrate superior chemical stability and resistance to oxidation, which enhances their long-term reusability in water treatment applications [54]. Kinetic studies further indicate that adsorption generally follows a pseudo-second-order model, suggesting chemisorption as the dominant mechanism, while elevated temperatures can improve overall efficiency [53,54].

Surface functionalization significantly improves the selectivity of MNPs for specific contaminants. Thiol-functionalized nanoparticles show enhanced uptake of soft metals such as cadmium and lead due to strong sulfur–metal interactions [55], while phosphate-modified Fe_3_O_4_ exhibits exceptional affinity for uranium and thorium, with sorption capacities exceeding 2300 mg U g^−1^ Fe, substantially higher than conventional adsorbents [56]. Additional modifications using poly(sodium acrylate) or cysteine provide further improvements via electrostatic and hard–soft acid–base interactions, facilitating the removal of metals like zinc, copper, and nickel from complex aqueous matrices [53]. These functionalization strategies not only enhance adsorption capacity and selectivity but also improve nanoparticle stability and recyclability under varied environmental conditions.

In South Africa’s mining-impacted regions, particularly those affected by acid mine drainage (AMD), MNPs offer distinct advantages due to their stability under acidic conditions and adaptability for field applications [57]. Recent advances in green synthesis, such as the use of rooibos tea extract, provide cost-effective and environmentally friendly routes to produce iron-based nanoparticles with strong catalytic and adsorption properties [58]. These approaches align with the sustainability needs of local communities, but challenges remain, including nanoparticle aggregation, synthesis costs, and uncertainties around ecotoxicological impacts if released into the environment [59]. Hybrid systems that integrate magnetic nanoparticles with polymeric supports, ceramic membranes, biochar, or plant-derived reducing agents are emerging as promising solutions, offering improved structural stability, easier recovery, enhanced contaminant selectivity, and greater compatibility with continuous-flow treatment systems in low-resource mining environments.

From a practical perspective, magnetic nanoparticles represent one of the most mature nanomaterial platforms for mining-water remediation because they combine high adsorption capacity with rapid magnetic recovery and relatively straightforward regeneration. Compared with many other nanomaterial classes, they offer the practical advantage of rapid magnetic recovery without requiring external light sources or complex solid–liquid separation processes. Nevertheless, their long-term performance may be compromised by particle aggregation, surface oxidation, and reduced adsorption efficiency in chemically complex waters characterized by high ionic strength and competing ions. These limitations have stimulated the development of functionalized magnetic nanocomposites and hybrid treatment systems designed to improve stability, selectivity, and field applicability under realistic mining conditions. Recent evidence from real mine-water systems further demonstrates the applicability of magnetic nanoparticles, with O’Neill et al. [60] reporting effective Zn removal from complex circumneutral mine waters despite the presence of competing ions. Although laboratory-scale performance is well established, additional pilot-scale investigations are required to validate long-term operational stability, regeneration efficiency, and economic feasibility under realistic mining-water conditions.

### 6.2. Metal Oxide Nanoparticles (TiO_2_, ZnO, MnO_2_, CeO_2_)

Metal oxide nanoparticles such as titanium dioxide (TiO_2_), zinc oxide (ZnO), manganese dioxide (MnO_2_), and cerium oxide (CeO_2_) have been widely studied for water treatment because of their high surface reactivity, tunable redox properties, and relative cost-effectiveness. TiO_2_ is widely applied for the removal of inorganic contaminants and organic pollutants, while ZnO exhibits comparable treatment potential together with antibacterial activity [61,62]. MnO_2_ and CeO_2_ have also demonstrated affinity for radionuclides and heavy metals, including uranium and arsenic [63]. These properties make metal oxide nanoparticles versatile candidates for the treatment of chemically complex contaminated waters. The physicochemical mechanisms responsible for adsorption, photocatalytic transformation, and redox-based contaminant removal are discussed separately in Section 7. Surface modification, doping, and composite formation have been widely investigated to improve the treatment performance of metal oxide nanoparticles. Modified TiO_2_ and ZnO systems have demonstrated improved activity under visible-light conditions, while functionalized MnO_2_- and CeO_2_-based materials have shown enhanced contaminant uptake and selectivity [63,64,65,66]. More recent developments, including heterojunction and high-entropy oxide systems, have further expanded the potential applicability of metal oxides in multi-contaminant water treatment [67]. Detailed photocatalytic and redox mechanisms associated with these modifications are discussed in Section 7.3.

In South Africa, where acid mine drainage (AMD) and radionuclide-rich effluents from the Witwatersrand Basin remain pressing concerns, metal oxides offer significant potential because of their performance under acidic and chemically complex conditions. TiO_2_-based treatment has achieved arsenic removal efficiencies above 87% under optimized AMD conditions, although challenges remain with catalyst recovery and external light requirements [68]. MnO_2_- and CeO_2_-based materials have also demonstrated potential for uranium and arsenic removal, although aggregation and reduced performance in multi-contaminant waters remain important limitations [69]. Hydrated ZrO_2_-based materials have similarly demonstrated high removal performance for metals in AMD-impacted waters [70]. Collectively, these findings support the potential application of metal oxides in mining-water remediation if limitations related to recovery, stability, and scalability are adequately addressed.

From a practical perspective, metal oxide nanoparticles provide a versatile platform for mining-water remediation because of their broad contaminant-removal capability and adaptable physicochemical properties. Compared with magnetic nanoparticles, metal oxides generally offer broader catalytic functionality but often require more complex recovery strategies, particularly when used as dispersed photocatalysts. Their performance is also strongly influenced by light availability, solution chemistry, and catalyst deactivation under prolonged operation. Consequently, increasing research has focused on immobilized photocatalysts, heterojunction composites, and hybrid treatment systems that improve catalyst recovery, operational stability, and long-term applicability in continuous-flow treatment of mining-impacted waters. Although numerous laboratory studies have demonstrated high contaminant removal efficiencies, further pilot-scale investigations are required to evaluate long-term durability, regeneration, and cost-effectiveness under realistic mining conditions.

### 6.3. Metal-Organic Frameworks (MOFs)

Metal–Organic Frameworks (MOFs) have emerged as one of the most versatile classes of porous nanomaterials for radionuclide and heavy metal removal because of their exceptionally high surface areas, tunable pore structures, and chemically functionalizable frameworks [71,72]. The unique architecture of MOFs allows for precise control of pore size and surface functionality, enabling efficient adsorption of radionuclides such as uranium (U), radium (Ra), and thorium (Th). Functionalization of MOFs with specific chemical groups, such as phosphonate, carboxylate, and amine, enhances their binding affinity and selectivity for these radionuclides, facilitating targeted interactions at the molecular level [72].

Recent developments have focused on composite and hybrid MOFs, where nanoparticles or carbon-based materials are incorporated to improve chemical stability and enhance adsorption efficiency [73,74]. These hybrids combine the structural advantages of MOFs with the durability of inorganic components, expanding their application potential beyond laboratory settings. For instance, modified MOFs have demonstrated high sorption capacities for uranium under mildly acidic conditions and retain structural integrity after multiple adsorption–desorption cycles, underscoring their reusability [71].

Compared with magnetic nanoparticles and metal oxide nanoparticles, MOFs generally provide higher surface areas and greater opportunities for selective adsorption through precise control of pore architecture and surface chemistry. However, these advantages are often offset by higher synthesis costs, greater sensitivity to aqueous and acidic environments, and more complex manufacturing processes. Consequently, the choice between MOFs and other nanomaterials depends on the specific treatment objective, contaminant characteristics, and operational conditions, with MOFs being particularly attractive where high selectivity for radionuclides or specific heavy metals is required.

However, stability and cost remain major barriers to widespread implementation. Many MOFs degrade under aqueous or acidic conditions, limiting their use in environments such as acid mine drainage (AMD). Post-synthetic modifications and the use of robust linkers, such as zirconium or aluminum-based frameworks, have shown promise in improving water stability and regeneration potential [72]. In South Africa, where AMD and radionuclide-rich effluents are prevalent in mining areas, the adaptability of MOFs offers potential for localized remediation strategies. Their tunable chemistry enables the rational design of adsorbents capable of selectively targeting uranium, radium, and other radionuclides prevalent in the Witwatersrand Basin, thereby supporting site-specific remediation strategies for mining-impacted water systems [71].

From a practical perspective, MOFs represent one of the most selective nanomaterial platforms for radionuclide and heavy metal removal because of their highly tunable pore structures and surface chemistry. Nevertheless, their widespread implementation in mining-water treatment remains constrained by synthesis costs, hydrolytic stability, and large-scale production challenges. Current research increasingly focuses on water-stable zirconium-based frameworks, green synthesis approaches, and MOF-based composites that improve durability while reducing production costs. Although laboratory studies have demonstrated excellent adsorption performance, additional pilot-scale investigations are needed to evaluate long-term stability, regeneration efficiency, and economic feasibility under realistic mining conditions.

### 6.4. Nanoclays and Zeolites

Nanoclays and zeolites, both natural and synthetic, have gained considerable attention for the remediation of radionuclide and heavy-metal-contaminated water due to their ion-exchange capacity, large surface area, and structural stability. These materials effectively remove contaminants such as uranium (U), lead (Pb), cadmium (Cd), cesium (Cs), and strontium (Sr) through ion exchange, surface complexation, and physical adsorption mechanisms [75]. Natural zeolites are abundant, inexpensive, and widely available, making them particularly suitable for large-scale remediation of mining-impacted waters. Their adsorption capacity and selectivity can be further enhanced by surface modification with surfactants or polymers, which improve affinity for both cationic and anionic metal species [75].

Synthetic zeolites, such as LTL and MOR types, exhibit even higher performance, achieving removal efficiencies of 95–99% for Pb^2+^, Cd^2+^, Cs^+^, and Sr^2+^ due to their tunable pore structures and high cation-exchange capacity [76]. Functionalization approaches, including guanidine or amine modification, enhance their selectivity for uranium and other oxyanions while also imparting antibacterial properties that are advantageous in complex natural waters [77]. Similarly, thermal activation of natural zeolites improves chemisorption of Cs^+^ and Sr^2+^, critical for applications in nuclear-waste and mine-water management [78]. Emerging techniques such as 3D printing of zeolite monoliths have further improved reusability, allowing these structures to maintain adsorption capacity and mechanical integrity across multiple treatment cycles.

Compared with magnetic nanoparticles, metal oxide nanoparticles, and MOFs, nanoclays and zeolites offer the advantages of lower material costs, greater natural abundance, and well-established ion-exchange properties, making them particularly suitable for large-scale water treatment applications. However, depending on the target contaminant and water chemistry, their adsorption capacities and selectivity may be lower than those of highly engineered nanomaterials, often necessitating surface modification or incorporation into hybrid composites to achieve comparable performance. Consequently, nanoclays and zeolites are especially attractive where cost-effectiveness, operational simplicity, and scalability are prioritized over maximum adsorption capacity.

In South Africa, the abundance of natural zeolitic and clay minerals presents an economically viable opportunity for addressing acid mine drainage and metal pollution. Locally sourced zeolites offer low-cost, scalable solutions that align with sustainable remediation goals by minimizing the need for imported treatment materials and enabling material regeneration and reuse, thereby reducing the environmental footprint of remediation processes [75]. Integrating these naturally available adsorbents into existing treatment frameworks could significantly enhance the long-term management of contaminated water systems in mining regions while supporting affordable, locally sourced remediation technologies that align with circular economy and sustainable resource management principles.

From a practical perspective, nanoclays and zeolites remain among the most economically attractive adsorbents for mining-water remediation because they combine low cost, natural abundance, chemical stability, and relatively simple regeneration. Although their adsorption capacities may be lower than those of highly engineered nanomaterials such as MOFs, their scalability and demonstrated applicability in large-volume treatment systems make them particularly suitable for resource-constrained mining regions. Future research should focus on advanced surface functionalization, hybrid nanocomposites, and pilot-scale evaluations to improve selectivity, regeneration efficiency, and long-term performance under complex mining-water conditions.

### 6.5. Biogenic/Green Nanoparticles

The green or biogenic synthesis of nanoparticles represents an environmentally sustainable alternative to conventional chemical and physical synthesis methods. This approach utilizes biological materials such as plants, algae, fungi, and bacteria to reduce and stabilize metal ions into nanoscale structures [79]. Plant extracts contain a variety of secondary metabolites, including flavonoids, alkaloids, terpenoids, and phenolic compounds, that act as natural reducing and capping agents, eliminating the need for toxic chemicals or high-energy processes [80,81]. The resulting nanoparticles often exhibit diverse morphologies and surface chemistries that influence their adsorption capacity, catalytic activity, and environmental stability.

Compared with traditional synthesis routes, green synthesis offers distinct advantages, including lower toxicity, minimal environmental impact, and cost-effectiveness [82]. These nanoparticles can be tailored for specific environmental applications by controlling parameters such as extract concentration, pH, and temperature. Various plants, including *Moringa oleifera*, *liana*, aloe, tea, and neem, have been successfully employed in producing metal and metal-oxide nanoparticles, demonstrating a capacity to remove heavy metals such as lead, cadmium, and arsenic from water systems [81,83]. Microbial routes using bacteria and fungi offer additional benefits, such as intracellular synthesis and better particle uniformity, though maintaining culture stability remains challenging [84].

In South Africa, green synthesis presents a promising pathway for developing low-cost, locally adaptable remediation technologies. Indigenous plant species and agricultural wastes have been identified as viable precursors for nanoparticle synthesis, providing an environmentally friendly route for the adsorption of heavy metals and the remediation of contaminated water [85]. Despite challenges related to large-scale production, reproducibility, long-term stability, and quality control, continued research on the functionalization and hybridization of biogenic nanoparticles could substantially enhance their adsorption performance, durability, and practical applicability in mining-water remediation [86]. As a sustainable and community-oriented technology, biogenic nanoparticles align with circular-economy principles and offer an accessible solution for mitigating mining-related water pollution in South Africa and similar regions.

From a practical perspective, biogenic nanoparticles represent one of the most sustainable approaches to nanotechnology-based water remediation because they integrate environmentally benign synthesis methods with effective contaminant removal. Compared with highly engineered nanomaterials such as MOFs and functionalized magnetic nanoparticles, they generally offer lower production costs and reduced environmental impacts but may require further optimization to achieve comparable adsorption efficiencies and long-term operational stability. Future research should prioritize standardized synthesis protocols, life-cycle assessments, pilot-scale demonstrations, and the development of hybrid biogenic nanocomposites to support commercialization and large-scale implementation in mining-impacted regions. The key characteristics, advantages, limitations, and practical applicability of the nanomaterial classes discussed in this section are summarized in Table 3.

## 7. Mechanisms of Radionuclide and Heavy Metal Removal by Nanomaterials

The removal of radionuclides and heavy metals by nanomaterials involves several interrelated mechanisms, primarily ion exchange, surface complexation, chemisorption, and photocatalytic redox reactions. The dominant removal pathway is strongly dependent on nanomaterial composition and surface chemistry, as well as environmental parameters such as pH, ionic strength, and temperature. This section focuses on the generalized removal mechanisms governing radionuclide and heavy metal interactions with nanomaterials, including ion exchange, surface complexation, physisorption, chemisorption, and photocatalytic redox reactions [88].

### 7.1. Ion Exchange and Surface Complexation

Ion exchange and surface complexation represent the primary adsorption-driven mechanisms through which nanomaterials immobilize radionuclides and heavy metals in aqueous systems, with the dominant pathway determined by nanomaterial composition and surface chemistry. In aluminosilicate-based nanomaterials such as zeolites and nanoclays, ion exchange governs contaminant removal through electrostatic substitution within a negatively charged lattice. Exchangeable framework cations, including Na^+^, K^+^, and Ca^2+^, are replaced by dissolved metal ions such as Pb^2+^, Cd^2+^, Cs^+^, and UO_2_^2+^ to maintain charge neutrality. This process is rapid and reversible, with selectivity controlled by ion valence, hydrated ionic radius, and solution chemistry, making ion exchange particularly effective for mono- and divalent metals commonly encountered in mining-impacted waters [76]. The high cation exchange capacity and well-defined microporous structure of zeolites further enhance their suitability for metal immobilization, while the rapid and reversible nature of ion exchange enables regeneration and reuse under controlled conditions [75,89].

In contrast, metal oxide nanomaterials, including iron oxides (Fe_3_O_4_, Fe_2_O_3_), manganese oxides (MnO_2_), cerium oxide (CeO_2_), and titanium dioxide (TiO_2_), remove radionuclides and heavy metals predominantly through surface complexation mechanisms. This process involves the formation of inner-sphere coordination bonds between dissolved metal species and hydroxylated surface sites, resulting in stronger and more chemically stable binding than ion exchange. For example, Pb^2+^ removal by Fe_3_O_4_ nanoparticles occurs through the formation of Fe–O–Pb surface complexes that are resistant to desorption, enabling sustained immobilization under variable aqueous conditions [90,91]. Oxyanion-forming species such as U(VI) and As(V) similarly exhibit strong affinity for oxide surfaces via ligand exchange reactions, as demonstrated for iron oxides interacting with arsenate and chromate [92]. The efficiency of surface complexation is strongly influenced by solution pH and ionic strength, with inner-sphere binding becoming dominant under conditions that favor surface hydroxyl deprotonation, as observed for Pb(II) adsorption on TiO_2_ surfaces [90]. The high surface reactivity and structural flexibility of nanoscale iron and manganese oxides further enhance their capacity to immobilize a broad range of contaminants through stable chemisorptive interactions [93].

Together, ion exchange and surface complexation define complementary adsorption pathways: ion exchange enables rapid uptake in aluminosilicate materials, whereas surface complexation on metal oxides provides strong, largely irreversible binding. Hybrid systems that combine these materials can exploit both mechanisms simultaneously, enhancing removal efficiency and robustness in chemically complex, multi-contaminant mining waters.

### 7.2. Physisorption Versus Chemisorption

Physisorption and chemisorption represent fundamentally different adsorption pathways that govern the stability and reversibility of contaminant uptake by nanomaterials. Physisorption is driven by weak electrostatic interactions, hydrogen bonding, and van der Waals forces, enabling rapid but reversible uptake of metal ions on high surface area materials such as unmodified clays and carbonaceous supports [94]. However, because these interactions do not involve chemical bond formation, physisorbed species are highly sensitive to changes in pH, ionic strength, and competing ions, making desorption likely under the fluctuating geochemical conditions typical of mining-impacted waters [95]. In contrast, chemisorption involves the formation of strong inner-sphere bonds, often covalent or coordination in nature, between contaminants and reactive surface sites, resulting in significantly greater resistance to desorption and enhanced long-term immobilization [96]. For example, Pb^2+^ and Cd^2+^ form stable metal–oxygen bonds on iron oxide surfaces, while U(VI) and As(V) bind through ligand exchange with hydroxylated oxide sites, resulting in higher adsorption energies and reduced reversibility compared to physisorption. This distinction is critical for remediation design, as physisorption is better suited for short-term or reversible uptake, whereas chemisorption provides the stability required for sustained containment of radionuclides and heavy metals in dynamic aquatic systems.

### 7.3. Photocatalytic Reduction Oxidation

Photocatalytic reduction and oxidation provide an important complementary mechanism for the removal of radionuclides and redox-sensitive heavy metals using semiconductor nanomaterials such as titanium dioxide (TiO_2_), zinc oxide (ZnO), and cerium oxide (CeO_2_). Upon irradiation with UV or visible light, these materials generate electron hole pairs that drive redox reactions capable of transforming contaminants into less mobile or less toxic forms. In particular, photogenerated electrons can reduce soluble U(VI) to sparingly soluble U(IV), while photogenerated holes oxidize As(III) to As(V), which exhibits stronger affinity for mineral surfaces and reduced mobility in aquatic systems. The efficiency of these transformations is governed by band-gap energy, charge-carrier recombination rates, surface area, and light availability. Strategies such as heterojunction formation and metal or non-metal doping have been shown to enhance photocatalytic performance by extending light absorption into the visible range and improving charge separation [64]. However, in mining-impacted waters, practical limitations including high turbidity, limited light penetration, and catalyst recovery constrain standalone photocatalytic applications. Consequently, photocatalysis is most effective when integrated with adsorption-based mechanisms, where redox transformation reduces contaminant mobility and adsorption ensures stable immobilization under complex geochemical conditions [97].

### 7.4. Nanoparticle Aggregation and Stability Constraints in Mining Waters

Despite their high reactivity, the practical deployment of nanomaterials for radionuclide and heavy metal remediation is limited by aggregation and stability challenges, particularly under the high ionic strength, variable pH, and complex geochemistry characteristic of mining-impacted waters. Charge screening by dissolved ions suppresses electrostatic repulsion between nanoparticles, promoting aggregation, reducing accessible surface area, and diminishing adsorption or catalytic efficiency [13,98]. Metal oxide and magnetic nanoparticles are especially susceptible to these effects under acidic or saline conditions. Natural organic matter can exert dual influences, either stabilizing nanoparticles through steric hindrance or enhancing aggregation via cation-bridging interactions in the presence of divalent ions such as Ca^2+^ [98,99]. To address these limitations, surface functionalization and immobilization onto solid supports such as clays, biochar, or membranes are increasingly employed to enhance colloidal stability, facilitate recovery, and reduce unintended nanoparticle release. Controlling aggregation and environmental stability is therefore essential for translating nanomaterial-based remediation from laboratory studies to scalable, field-applicable solutions in mining-affected water systems. A comparative summary of nanomaterial classes, dominant removal mechanisms, target contaminants, and key advantages and limitations in mining-impacted water systems is presented in Table 4.

## 8. Comparative Analysis and Future Directions

### 8.1. Comparative Performance and Application of Nanomaterials

The nanomaterials reviewed in this study exhibit distinct strengths and limitations when evaluated in terms of removal efficiency, selectivity, stability, and suitability for mining-impacted water systems. Magnetic nanoparticles offer high adsorption capacities and the advantage of facile magnetic recovery, but their effectiveness is frequently constrained by aggregation and sensitivity to variations in water chemistry, which can undermine stability under field conditions [100]. Metal oxide nanomaterials provide strong chemisorptive binding and redox functionality, enabling stable immobilization of radionuclides and heavy metals; however, challenges related to catalyst recovery and long-term environmental stability may limit their practical deployment [101]. In contrast, nanoclays and zeolites are cost-effective, naturally abundant, and scalable, yet they generally exhibit lower selectivity and reduced performance in systems containing complex or redox-active contaminants [102]. Emerging materials such as metal–organic frameworks demonstrate exceptional selectivity at the laboratory scale, but issues of water stability, regeneration, and cost currently hinder their broader environmental application [103]. Biogenic and green-synthesized nanoparticles represent a sustainable alternative, though further validation is required to ensure reproducibility, durability, and effectiveness in real mining waters [104]. Collectively, these comparisons highlight that successful remediation strategies must balance removal efficiency with stability, scalability, and site-specific water chemistry to enable reliable field implementation.

### 8.2. Trade-Offs Between Efficiency, Stability, and Scalability

The application of nanomaterials in mining-impacted water systems reveals a fundamental trade-off between high removal efficiency and practical constraints related to stability, scalability, and operational feasibility. Engineered nanomaterials such as metal oxides and metal organic frameworks often exhibit exceptional selectivity and adsorption capacity under controlled laboratory conditions; however, their performance frequently declines in real waters characterized by high ionic strength, variable pH, competing ions, and suspended solids [13,105]. Magnetic nanoparticles similarly enable efficient contaminant uptake and recovery, yet aggregation and surface passivation can compromise long-term effectiveness unless stabilization strategies are implemented [106]. In contrast, naturally abundant materials such as nanoclays and zeolites offer greater robustness and scalability but typically sacrifice selectivity and performance toward redox-active or low-concentration radionuclides [107]. These trade-offs highlight that remediation efficiency alone is insufficient as a performance metric; material stability, regeneration potential, cost, and compatibility with site-specific water chemistry must also be considered. Consequently, hybrid and composite systems that integrate complementary functions, such as adsorption, redox transformation, and physical immobilization, are increasingly regarded as the most viable pathway toward scalable and resilient remediation strategies for mining-impacted waters [70].

### 8.3. Laboratory-to-Field Transition: Knowledge Gaps and Key Failure Factors

The translation of nanomaterial-based remediation from laboratory studies to field-ready applications in mining-impacted water systems remains constrained by several critical knowledge gaps. A major limitation is the insufficient evaluation of nanomaterials under realistic environmental conditions, where complex water chemistries, characterized by high ionic strength, variable pH, and mixed radionuclide metal contamination, can substantially alter material performance. For example, the aggregation, surface passivation, and loss of reactivity of zerovalent iron and metal oxide nanoparticles are strongly influenced by ionic strength and pH, yet these factors are often inadequately represented in laboratory experiments [13,108]. Long-term stability and transformation processes, including aging and irreversible aggregation, remain poorly understood, particularly in systems containing multiple contaminants. In addition, regeneration efficiency and material reuse are typically assessed under simplified conditions, limiting insight into lifecycle performance and economic feasibility [109]. Ecotoxicological impacts and the environmental fate of released or transformed nanoparticles also remain underexplored, raising concerns regarding secondary contamination and regulatory acceptance [110]. Furthermore, many studies prioritize removal efficiency as the primary metric, while neglecting scalability, material recovery, and compatibility with existing treatment infrastructure [111]. Addressing these challenges requires standardized testing using real mine waters, long-term pilot-scale investigations, and interdisciplinary approaches that integrate materials science, environmental chemistry, and risk assessment to enable the development of safe, resilient, and deployable nanotechnology-based remediation strategies [13].

### 8.4. Future Pathway Toward Field Deployment

Building on the laboratory-to-field barriers identified in Section 8.3, future progress should shift from proof-of-concept performance toward practical deployment strategies that emphasize durability, scalability, and system-level integration. Composite and immobilized nanomaterials, such as nanoparticles anchored onto clays, biochar, membranes, or granular substrates, represent a promising pathway for facilitating material recovery and enabling continuous-flow operation. Future investigations should increasingly evaluate these systems at pilot scale under realistic treatment volumes and operating conditions [112].

Integration of nanomaterials into existing treatment infrastructure is another critical direction for future research. Hybrid systems that couple nanomaterial-based adsorption or redox processes with conventional treatment units (e.g., filtration, passive treatment, or constructed wetlands) may offer more practical and cost-effective solutions than standalone nanotechnology platforms. Such approaches are particularly relevant for mining regions in low- and middle-income settings, where operational simplicity, low energy demand, and minimal maintenance are essential for long-term adoption [74].

Advances in green synthesis and locally sourced nanomaterials also present opportunities to align remediation technologies with sustainability and circular-economy principles. The use of plant-based precursors, industrial by-products, or mine-derived materials for nanoparticle synthesis could reduce costs and environmental footprints while improving social acceptance [104]. However, these approaches must be accompanied by rigorous assessments of reproducibility, long-term stability, and environmental safety to ensure reliable field performance [13].

Finally, future research should prioritize standardized evaluation frameworks that incorporate real mine waters, long-term exposure, life-cycle assessment, and techno-economic analysis, alongside regulatory and ecotoxicological considerations. Collaboration between materials scientists, environmental engineers, geochemists, and policymakers will be essential to bridge the gap between laboratory innovation and field implementation. By prioritizing deployability, cost-effectiveness, environmental safety, and long-term operational performance rather than material novelty alone, nanotechnology has the potential to evolve from an experimental research tool into a practical and sustainable solution for improving water quality in mining-impacted regions.

## 9. Conclusions

This review critically examined the current state of nanotechnology-based strategies for mitigating radionuclide and heavy metal contamination in mining-impacted water systems, with particular emphasis on contaminant removal mechanisms, material performance, and prospects for field deployment. Beyond graphene-based materials, nanomaterial classes such as magnetic nanoparticles, metal oxides, nanoclays, zeolites, metal organic frameworks, and biogenic nanoparticles exhibit distinct yet complementary removal pathways, including ion exchange, surface complexation, chemisorption, and photocatalytic redox transformation. The comparative analysis demonstrates that no single nanomaterial provides a universal solution for all mining-water treatment scenarios. Instead, material selection should be site-specific and guided by contaminant speciation, water chemistry, pH, ionic strength, competing ions, operational conditions, and treatment objectives. Mechanistic understanding therefore emerges as a critical foundation for selecting appropriate nanomaterials and designing efficient remediation systems capable of addressing the complex chemistry of mining-impacted waters.

From an application perspective, the transition of nanotechnology from laboratory-scale success to field-ready remediation remains constrained by complex mine-water chemistry, nanoparticle aggregation and surface passivation, material recovery, regeneration, long-term stability, scalability, and compatibility with existing treatment infrastructure. These factors can reduce the performance observed under controlled laboratory conditions and therefore represent major barriers to reliable field implementation. Composite and immobilized nanomaterials, green synthesis routes, and hybrid treatment systems that integrate nanomaterials with existing infrastructure represent promising pathways toward practical deployment.

Future research should prioritize standardized evaluation protocols using real mining waters, long-term performance assessment, life-cycle assessment, techno-economic analysis, and pilot-scale validation alongside removal efficiency. By shifting the emphasis from material novelty toward site-specific suitability, durability, deployability, environmental safety, and long-term operational performance, nanotechnology has the potential to become a practical and sustainable component of water-treatment strategies for mining-impacted regions.

## Figures and Tables

**Table 1:** Reported concentrations of radionuclides and heavy metals in mining impacted waters compared with international drinking water and environmental guideline limits.

Contaminant	Mining Context	Mining Region	Reported Concentration Range	Guideline Limit	Comparison with Guideline	Guideline Source	References
Uranium (U)	Gold and uranium mining,	Rietspruit, Far West Rand, Goldfield, Gauteng Province, South Africa	0.3–781.9 µg L^−1^	30 µg L^−1^	Range includes concentrations exceeding guideline	[28]	[25]
Radium (^226^Ra)	Uranium mine tailings drainage	Gold and uranium mining	0.00097–0.0195 Bq L^−1^	1 Bq L^−1^	Below guideline	[28]	[1]
Arsenic (As)	Groundwater near mine tailings	Crocodile River (West) system, Gauteng, South Africa; Nigerien Liptako, Southwestern Niger	0.018–202 µg L^−1^	10 µg L^−1^	Range includes concentrations exceeding guideline	[28]	[23,29]
Lead (Pb)	Lead–zinc mining	Abakaliki, Southeast Nigeria	0–420 µg L^−1^	10 µg L^−1^	Range includes concentrations exceeding guideline	[28]	[3]
Nickel (Ni)	Artisanal and small-scale gold mining	Kakamega and Vihiga Counties, western Kenya	0.9–218 µg L^−1^	20 µg L^−1^ for drinking water	Concentrations exceeded guideline at some sites	[28]	[30]
Cadmium (Cd)	Zn–Sn mining and artisanal mining	Riruwai Mining Area, Kano State, Northern Nigeria	1–150 µg L^−1^	3 µg L^−1^	Range includes concentrations exceeding guideline	[28]	[31]
Mercury (Hg)	Artisanal and traditional gold mining	Migori Gold Belt, Lake Victoria Basin, Kenya; Paningkaban Village, Gumelar, Banyumas Regency, Indonesia	0.36–12,354.4 µg L^−1^	6 µg L^−1^	Range includes concentrations exceeding guideline	[28]	[32,33]

**Table 2:** Conventional water treatment technologies for radionuclide and heavy metal removal: operational principles, advantages, and limitations in mining-impacted contexts.

Treatment Technology	Operational Principle	Target Contaminants	Reported Removal Efficiency	Energy/Cost Considerations	Advantages	Limitations	References
Aeration	Velarization and gas transfer of dissolved radon from water to air	^222^Rn	35–99.9%, depending on aeration configuration; PTA: 90–99%	Energy and infrastructure requirements vary with aeration configuration; quantitative energy/cost values not consistently reported	Effective for dissolved radon; rapid treatment; packed-tower aeration can achieve very high removal	Limited effectiveness for dissolved metals; energy-intensive operation and potential secondary contamination	[45,49]
Granular Activated Carbon (GAC)	Adsorption of radon onto porous activated-carbon surfaces	^222^Rn	50–99%, depending on operating conditions	Relatively simple operation; carbon replacement/regeneration contributes to operating cost; standardized energy/cost values not reported	Widely available, established	Accumulation of radioactive decay products such as ^210^Pb and ^210^Po; spent carbon requires appropriate handling and disposal	[45,49]
Chemical precipitation	Conversion of dissolved metal ions into insoluble precipitates, followed by separation	Heavy metals, including Cu, Mn, Cd and Zn	Up to ~100% under optimal conditions (pH 4.0; HRT 24 h) in an AMD treatment system	Generally simple and relatively low-cost; passive AMD systems can have low operating costs; standardized energy values not reported	Effective for high metal concentrations; relatively simple operation; applicable to AMD	Performance depends on pH and treatment conditions; precipitate/sludge generation requires management; solid–liquid separation may be required	[50,51]
Ion exchange (resins)	Ionic substitution	Dissolved ionic radionuclides and heavy metals	High but contaminant- and material-specific; no universal efficiency value	Regeneration/replacement of exchange media contributes to operating cost; standardized energy/cost values not reported	High selectivity; simple operation; suitable for dissolved ionic contaminants	Performance affected by salinity and competing ions; regeneration can be difficult; saturation and secondary radioactive waste generation	[46,49]
Reverse osmosis/nanofiltration (RO/NF)	Size and charge-based separation through semi-permeable membranes	Dissolved heavy metals, radionuclides, salts and other ionic contaminants	~95–98% rejection reported for AMD contaminants/heavy metals	NF operates at lower pressure than RO and can reduce energy demand; treatment cost depends on pressure, pretreatment and membrane maintenance	High contaminant rejection; broad-spectrum removal; produces high-quality treated water	Severe fouling under AMD conditions, high energy demand, concentrate disposal, and operational cost	[20,52]

**Table 3:** Comparative evaluation of nanomaterial classes for radionuclide and heavy metal remediation in mining-impacted water systems.

Nanomaterials	Primary Removal Mechanism	Typical Target Contaminants	Removal Performance	Operational pH	Performance Under AMD Conditions	Regeneration Potential	Field Readiness	Major Limitations	Key References
Magnetic nanoparticles (Fe_3_O_4_, CoFe_2_O_4_)	Surface complexation, electrostatic attraction	Pb(II), Cd(II), Zn(II), Cu(II), Ni(II), Cr(III/VI)	>90% to ~100% removal reported for several metals under optimized conditions	Near neutral (~5–7), contaminant-dependent	Good, but high ionic strength and low pH may promote aggregation and reduce performance	High	Pilot-scale demonstrated	Aggregation, oxidation, recovery cost	[53,54,55,56,57,58,59]
Metal oxides (TiO_2_, ZnO, MnO_2_, CeO_2_)	Adsorption, photocatalysis, redox	U, As, Pb, organics	High (As removal >87%; material dependent)	Acidic–neutral	Moderate–Good	Moderate	Pilot-scale/application evidence remains material-specific	Light requirement, aggregation, recovery	[61,62,63,64,65,66,67,68,69,70,87]
MOFs	Coordination chemistry, adsorption	U, Ra, Th, Pb	Very high (material dependent)	Mildly acidic–neutral	Moderate; stability can decrease under complex aqueous conditions	Moderate-High	Primary laboratory-scale	Hydrolytic instability, synthesis cost and variable recyclability	[71,72,73,74]
Nanoclays & Zeolites	Ion exchange, adsorption	U(VI), Cs^+^, Sr^2+^ and heavy metals	Moderate–High (95–99% for selected metals)	Wide	Good, although competing ions may reduce selectivity/capacity	High	Commercial and Pilot-scale	Lower selectivity for some oxyanions; competing ions reduce performance	[75,77,78]
Biogenic nanoparticles	Adsorption, reduction	Pb, Cd, As, U	Moderate–High (depends on biological precursor)	Variable	Moderate, evidence under real AMD conditions remains limited	Moderate	Emerging, predominantly laboratory-scale	Batch-to-batch reproducibility, precursor variability, long-term stability and scale-up	[80,82,84,85,86]

**Table 4:** Major mechanisms governing radionuclide and heavy metal removal by nanomaterials in mining-impacted waters.

Removal Mechanism	Mechanistic Basis	Representative Nanomaterials	Target Radionuclides /Heavy Metals	Key Influencing Factors	Practical Implications	References
Ion exchange	Replacement of exchangeable ions within negatively charged material structures by dissolved contaminant ions	Zeolites, nanoclays	Pb^2+^, Cd^2+^, U(VI), As(V)	Ion valence, hydrated ionic radius, competing ions, pH	Rapid and potentially reversible uptake; regeneration may be possible	[75,76,89]
Surface complexation	Formation of inner-sphere coordination bonds between contaminants and reactive surface hydroxyl groups		U(VI), As(V), Pb^2+^	pH, ionic strength, surface charge, contaminant speciation	Strong contaminant binding and stable immobilization	[90,91,92,93]
Physisorption	Weak electrostatic interactions, hydrogen bonding and van der Waals forces	Nanoclays and other high-surface-area materials	Heavy metals and other dissolved contaminants	pH, ionic strength, competing ions, surface area	Rapid and reversible uptake but greater susceptibility to desorption	[94,95]
Chemisorption	Formation of strong chemical or coordination bonds between contaminants and reactive surface sites	Iron oxides	Pb^2+^, Cd^2+^, U(VI), As(V)	Surface functional groups, pH, contaminant speciation	Stronger and less reversible immobilization than physisorption	[90,92,96]
Photocatalytic redox transformation	Light-induced electron—hole generation drives reduction or oxidation of redox-sensitive contaminants	TiO_2_, ZnO, CeO_2_ and modified semiconductor nanomaterials	U(VI), As(III), Cr(VI), organic pollutants	Light availability, band gap, charge recombination, pH, turbidity	Can transform contaminants into less mobile or less toxic forms; performance may be constrained in turbid waters	[64,97]

## Data Availability

No new data were created or analyzed in this study. Data sharing is not applicable to this article.

## Abbreviations

The following abbreviations are used in this manuscript:

AMD	Acid Mine Drainage
GAC	Granular Activated Carbon
RO	Reverse Osmosis
NF	Nanofiltration
MNPs	Magnetic Nanoparticles
MOFs	Metal-Organic Frameworks
GO	Graphene Oxide
rGO	Reduced Graphene Oxide
WHO	World Health Organization
Fe_3_O_4_	Magnetite
COFe_2_O_4_	Cobalt Ferrite
TiO_2_	Titanium Dioxide
ZnO	Zinc Oxide
MnO_2_	Manganese Dioxide
CeO_2_	Cerium Oxide
U	Uranium
Ra	Radium
Th	Thorium
Pb	Lead
Cd	Cadmium
As	Arsenic
Hg	Mercury
U(VI)	Hexavalent Uranium
U(IV)	Tetravalent Uranium
As(III)	Arsenite
As(V)	Arsenate
